# Integrating Curriculum-Based Dynamic Assessment in Computerized Adaptive Testing: Development and Predictive Validity of the EDPL-BAI Battery on Reading Competence

**DOI:** 10.3389/fpsyg.2018.01492

**Published:** 2018-08-28

**Authors:** Juan-José Navarro, Catalina Mourgues-Codern, Eduardo Guzmán, Isabel R. Rodríguez-Ortiz, Ricardo Conejo, Claudia Sánchez-Gutiérrez, Jesús de la Fuente, Diana Martella, Mahia Saracostti

**Affiliations:** ^1^Department of Psychology and Sociology, Universidad de Zaragoza, Zaragoza, Spain; ^2^Facultad de Educación, Universidad Autónoma de Chile, Santiago, Chile; ^3^Departamento de Psicología Evolutiva y de la Educación, Universidad de Sevilla, Seville, Spain; ^4^Laboratoire Adaptations-Travail-Individu, Université Paris Descartes, Paris, France; ^5^Departamento de Lenguajes y Ciencias de la Computación, Universidad de Málaga, Málaga, Spain; ^6^Spanish and Portuguese Department, University of California, Davis, Davis, CA, United States; ^7^Departamento de Psicología Evolutiva y de la Educación, University of Almería, Almería, Spain; ^8^Facultad de Psicología, Universidad Autónoma de Chile, Santiago, Chile; ^9^Centro de Investigación sobre Procesos Socioeducativos, Familias y Comunidades, Universidad de La Frontera, Temuco, Chile

**Keywords:** dynamic assessment, computerized adaptive testing, item response theory, learning potential, graduated prompts, reading processes, incremental validity

## Abstract

In recent decades there have been significant changes in the conceptualization of reading as well as in the perception of how this activity should be assessed. Interest in the analysis of reading processes has led to the emergence of new explanatory models based primarily on the contributions of cognitive psychology. In parallel, there have been notable advances in measurement procedures, especially in models based on Item Response Theory (IRT), as well as in the capacity and performance of specific software programs that allow data to be managed and analyzed. These changes have contributed significantly to the rise of testing procedures such as computerized adaptive tests (CATs), whose fundamental characteristic is that the sequence of items presented in the tests is adapted to the level of competence that the subject manifests. Likewise, the incorporation of elements of dynamic assessment (DA) as the prompts are gradually offered allows for obtaining information about the type and degree of support required to optimize the subject’s performance. In this sense, the confluence of contributions from DA and CATs offers a new possibility for approaching the assessment of learning processes. In this article, we present a longitudinal research developed in two phases, through which a computerized dynamic adaptive assessment battery of reading processes (EDPL-BAI) was configured. The research frame involved 1,831 students (46% girls) from 13 public schools in three regions of Chile. The purpose of this study was to analyze the differential contribution on reading competence of dynamic scores obtained in a subsample composed of 324 (47% girls) students from third to sixth grade after the implementation of a set of adaptive dynamic tests of morpho-syntactic processes. The results achieved in the structural equation modeling indicate a good global fit. Individual relationships show a significant contribution of calibrated score that reflects estimated knowledge level on reading competence, as well as dynamic scores based on the assigned value of graduated prompts required by the students. These results showed significant predictive values on reading competence and incremental validity in relation to predictions made by static criterion tests.

## Introduction

In educational contexts, assessing students’ cognitive skills and reading processes is central to making informed decisions about the support they require to reach their full potential. In this context, *Dynamic Assessment* (DA) has emerged as an alternative to traditional or “static" assessment methods and is better adapted to the detection of learning difficulties and special educational needs (Jitendra and Kameenui, 1993; Swanson and Lussier, 2001; Rezaee and Ghanbarpour, 2016). DA refers to a set of procedures that embeds intervention within the assessment process through feedback, guidance on the use of specific metacognitive processes, and mediation. It can also be achieved through a progressive sequence of explicit and targeted prompts. In the latter case, the degree of learning achieved by students when receiving such support is used as an indicator of learning potential.

In recent decades significant changes have also emerged in the conceptualization of specific domains of learning, such as reading and arithmetic. These new conceptualizations come hand in hand with new perceptions of how these activities should be evaluated. Specifically, in the context of reading development, new findings in cognitive psychology have contributed to the emergence of new explanatory models (Hacker et al., 2009; Thiede et al., 2009). This has happened in parallel with significant advances in measurement procedures, especially in relation to models based on Item Response Theory (IRT), and with a significant increase in the capacity and performance of specialized data analysis and data management software. These changes have contributed to the rise of computer-based adaptive assessment procedures, known as *Computerized Adaptive Testing* (CAT) (Embretson and Reise, 2000). In essence, their main feature is that the sequence of items presented in the test adapts to the estimated level of the student’s competency. However, the development of adaptive dynamic tests based on a mediation process has shown a lower degree of progress in the field of CAT, due in part to technical difficulties, but also to the complicated theoretical decisions that need to be taken regarding the type and intensity of the mediation provided by the system or the evaluator. Incorporating elements of DA, such as the prompts that are gradually offered to the students when they fail to correctly respond to an item, could increase the advantages of CAT. It is precisely this combination of DA and CAT that would situate these models in the realm of intervention-oriented evaluation. Indeed, when data are provided by the system on the resolution process followed by the student during a test, it is expected to be easier to infer patterns of successful intervention that directly address the specific issues observed during testing. Thus, instead of simply assessing the current state of students’ competencies, it also opens avenues for improvement based on the specific types of aids that better work for each student.

In this context, the current study aims to analyze the differential contribution on reading competence of the dynamic scores obtained from the implementation of a set of adaptive dynamic tests of morpho-syntactic processes integrated into the computerized adaptive DA battery EDPL-BAI. First, some key elements related to CATs and DA of reading competence is introduced. Then the design, structure, and content of the EDPL-BAI battery are presented.

### Computerized Adaptive Testing

**FIGURE 1:**
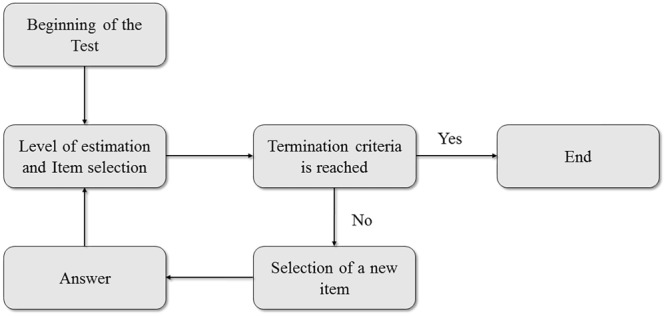
Sequence of actions in a computerized adaptive test.

Computerized adaptive testing proposes the progressive adaptation of the evaluated contents to the subject’s estimated abilities at each level of difficulty. Thus, the items presented to each student depend at all times on the student’s demonstrated ability during the execution of the task. The fundamental idea of CAT is to get as close as possible to the behavior that a human evaluator would demonstrate when trying to obtain information about a student’s task resolution abilities during a test (Wainer, 1990). The evaluator is expected to adapt questions to the answers that the student gives. If a question is too difficult for the student, he or she will most likely give a wrong answer, and therefore the evaluator will subsequently ask a question that is somewhat easier. The intention is to obtain the most accurate information possible about the student’s knowledge (van der Linden and Glas, 2000). CAT items are generally displayed one at a time, and decisions about the presentation of each item, the termination of the test and the evaluation process, in general, are made dynamically based on student responses. In essence, a CAT is thus an iterative algorithm that starts with an initial estimate of a student’s knowledge, which is usually represented by a probability distribution. Subsequently, all items are examined to determine which would be best suited to estimate the subject’s level of knowledge most accurately; the best fit is then selected, and the student responds. Based on the student’s answer, a new estimate of his or her knowledge is made, and a new item is selected that best fits the expected student’s knowledge level. This process continues until the termination criterion is reached or the set time is up, if there is a time limit. The student’s knowledge level is calculated as the mean or mode of the distribution calculated by this iterative process. **Figure 1** presents a typical CAT sequence.

**FIGURE 2:**
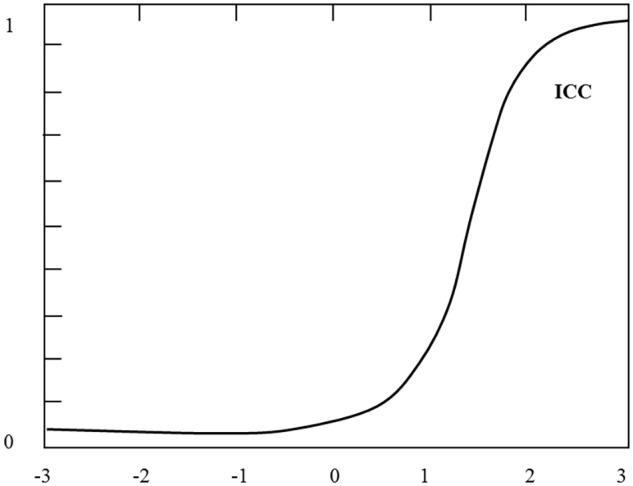
Item characteristic curve.

In the configuration of a CAT, a set of parameters that determine the characteristics of an evaluation session need to be taken into consideration. Among other relevant information, the following set of information should be specified: (a) the item bank (IB; i.e., the set of items from which the final selection will be drawn); (b) the criterion for initially estimating the level of a feature, such as the initial knowledge of the student; (c) the criterion for dynamically selecting items for the test (i.e., how to decide which item is going to be shown to the student at each trial); (d) the criterion for completion of the test (e.g., once the assessment of student knowledge has sufficient statistical accuracy, after a certain time, after completing all test items); and (e) the evaluation criteria (i.e., how the score is calculated). This is generally done by applying IRT methods, although there are also other options. For example, some heuristics could be used, such as the percentage of successful items or penalty points for each error. In short, most CATs’ item selection strategies are based on an estimate of the assessed latent trait based on each response from the student. To carry out such estimates it is necessary to calibrate all the items in the IB. Item calibration is a complex process by which an item characteristic curve (ICC) is inferred, such as the one shown in **Figure 2**. This curve represents the probability that an item will be correctly answered and is usually described by logistic functions of one (1PL), two (2PL), or three parameters (3PL) based on the following formula:

$$\begin{array}{c} P(u_{i}=1|\theta )=c_{i}+(1-C_{I})\frac{1}{1+e^{1.7a_{i}(\theta -b_{i})}} \end{array}$$

Where *P(u_i_ = 1|𝜃)* is the probability of correctly responding to item *i* given the student’s knowledge level *𝜃. P* is usually measured on a scale from -3.0 to 3.0. The three parameters that characterize this curve depend on the item and include the following: (1) *Discrimination* (*a_i_*) is a value that is proportional to the slope of the curve; the higher the value of this parameter, the better the item is at discerning between lower and upper levels of knowledge. (2) *Difficulty* (*b_i_*) corresponds to the knowledge level for which the probability of answering correctly is the same as that of answering incorrectly (regardless of random responses). (3) Finally, *Guess* or *chance* (*c_i_*) accurately measures the probability of correctly answering the question without the actual knowledge necessary to do so—that is, the probability of guessing at random. When ICC is determined by the model 1PL, the only parameter used is *difficulty*; *discrimination;* and *guessing*, in this case, both present a value of zero. In the 2PL model, besides *difficulty, discrimination* must also be estimated.

In order to perform the calibration process, a considerable volume of data about the items needs to be gathered from students who have completed them all. The calibration procedure is also an iterative algorithm that finds the parameters of the ICC (*difficulty, discrimination*, and/or *guessing*, depending on the model selected) that better explain the response matrix obtained by the sample of students who have participated in the calibration process. Once items are calibrated, the use of a CAT requires (1) an initialization procedure of the student knowledge distribution, depending on whether or not prior information about the subjects is available; (2) an algorithm to dynamically select items during the administration of the test, and (3) a completion criterion, following which the final estimate of the trait being evaluated can be computed.

After an item *i* has been presented to the student, and he or she has provided an answer, the student’s distribution of estimated knowledge, *P(𝜃| u_1…_, u_i_)*, is updated according to the following formula:

$$\begin{array}{c} P(\theta |u_{1},...,u_{i+1})\;=\;P(\theta |u_{1},...,u_{i})\;*\;P{\left(u_{i}\;=\;1|\theta \right)}^{u_{i}}\;*\;{\left[1-P(u_{i}\;=\;1|\theta )\right]}^{1-u_{i}} \end{array}$$

Where P(𝜃|u_1_,...,u_i+1_) is the distribution of student knowledge that has been updated after responding to item *i.* If the student’s answer is correct (*u_i_* = 1), the distribution is multiplied by the ICC; otherwise, it is multiplied by the opposite curve.

### Dynamic Assessment of Reading Processes

In the school context, DA results from the interaction between the evaluated student and a more proficient subject, usually an educator or psychologist, through a mediation process. This interactive process bridges the gap between the current competencies of the student and the demands of the task. As such, it creates opportunities for the joint construction of knowledge through interaction, which is especially relevant for psychoeducational interventions and the evaluation of learning processes (Newman et al., 1989; Vygotsky, 1995; Wells, 1999; Jensen, 2000; Sternberg and Grigorenko, 2002; Elliott, 2003). These mediated interactions facilitate the determination of the type and degree of support required by the students to successfully complete the tasks on which they are assessed and to acquire the established competencies. Incrementally, DA allows for establishing dynamic measures of the cognitive processes involved in task resolution, including those that are still under development (Sternberg and Grigorenko, 2002). Thus, data obtained through DA may contain relevant information that adds to that which can be obtained through more conventional static assessment methods. DA also offers more in-depth insights into the mechanisms of action that facilitate learning and task resolution in each specific student, which is necessary not only to support the students individually but also to offer better explanations of the improvement observed during an intervention. To date, various approaches to DA have been used to consider it as an assessment of (a) learning potential (Budoff, 1967), (b) test conditions (testing the limits) (Carlson and Wiedl, 2000), (c) mediated learning (Feuerstein et al., 1981), or (d) learning and assisted transfer (Campione et al., 1985). The latter approach, characterized by the use of graduated prompts during the evaluation process, has been one of the most widely used in developing computerized DAs. This is because the type and frequency of each prompt can be standardized and used as an indicator of the level of support that students need to learn a skill. For example, in relation to the type of support, one parameter may be the level of explicitness of support, making it possible to construct gradients of aid that are gradually more explicit and targeted, depending on the observed needs of each evaluated student.

Curriculum-based DA models include contextualized tasks that are closely related to educational content (Delclos et al., 1992; Ruijssenaars et al., 1993; Jensen, 2000; Guterman, 2002; Elliott, 2003; Kalyuga and Sweller, 2005; Swanson and Howard, 2005; Haywood and Lidz, 2007; Thurman and McGrath, 2008; Fuchs et al., 2011; Lidz, 2014). The adoption of such DA models could facilitate the incorporation of achievements reached during the assessment process into classwork (Jensen, 2000). In this sense, the aids that allowed the students to respond to higher-difficulty items during the assessment process could guide the type of intervention and support that the student needs. Thus, similar aids could be used in the classroom or in one-on-one intervention settings. Such contextualization endeavors, including collaboration with classroom teachers, should provide greater ecological validity to the process and results of DA.

Lately, DA models have been used in the specific field of learning difficulties related to reading with the purpose of obtaining profiles of learning potential and to establish the predictive value of dynamic tests on student achievement (Jeltova et al., 2007; Gustafson et al., 2014). In this regard, Caffrey et al.’s (2008) literature review showed that the predictive value of DA was higher than that of traditional evaluation methods when the level of achievement and support required to achieve that level were taken into consideration. In the last 2 years, more than 100 published studies have reported using DA for predicting reading difficulties in preschool students (Catts et al., 2015; Gellert and Elbro, 2015; King et al., 2015; Petersen et al., 2016), and in the early school years (Clemens et al., 2015; Fani and Rashtchi, 2015; Naeini, 2015; Wolter and Pike, 2015; Stevenson et al., 2016). For example, King et al. (2015) dynamically evaluated the production of sentence structures by 4- and 5-year-old children through graphic symbols on an augmentative and alternative communication (AAC) device. Incrementally, the predictive validity of the DA on a subsequent experimental task was evaluated. The four participants had normal receptive linguistic ability but presented limitations in speech production. Graduated prompts were used throughout the assessment procedure. The measures included the amount of support required to produce sentences using symbols, as well as the changes observed during the development of the sessions (modifiability). The authors showed that participants needed variable amounts of support to produce the target structures. Likewise, modifiability was more evident in some participants than others. With regard to the predictive validity of the assessment method, the results partially supported the predictive value of the dynamic test relative to the experimental work carried out later. The researchers concluded that DA yielded valuable information on the process followed by the participants to sequence simple messages based on rules through the AAC device.

Another study that focused on morpho-syntactic processes in the context of DA was conducted by Hasson et al. (2012). Twenty-four children between 8 and 10 years old participated. All of them had specific language impairments (SLIs). A DA method was designed that aimed to use the information obtained from the testing sessions to plan interventions that would specifically address the needs of children with SLI. The researchers argued that little is known about how individuals with SLI deal with the completion of language tasks, and that the use of static assessment has contributed to limiting our understanding of how children within this group address different types of linguistic skills. The DA procedure was applied four times for each participant, at intervals of 4 months between sessions. The predictive validity of the developed dynamic test was higher than that of the standardized test. The results of this study offered relevant insights into children’s abilities to use specific strategies, to take advantage of the guidelines provided during the testing sessions and to transfer learning from one item to the next. The authors concluded that the information obtained would be useful for speech therapists who plan specific interventions for children with SLI.

Dynamic assessment has been increasingly used in various fields, and this has been particularly true for some specific areas of knowledge, such as second language learning (Kozulin and Garb, 2002; Poehner, 2007, 2008; Ableeva, 2010; Lantolf and Poehner, 2011; Poehner et al., 2015). For example, Poehner et al. (2015) developed a computerized dynamic test that assessed reading and listening comprehension in a second language. The authors argued that mediation was essential in diagnosing the level of development reached by the learners. Each test item was accompanied by a set of prompts that were graduated from lower to higher explicitness. Thus, the final result of the evaluation included information not only about the questions that students correctly answered without assistance, but also about the amount of support needed during the resolution of each question. Poehner et al. (2015) used a heuristic to calculate the difference between the score without aids and the score that included data about the required aid. These scores, which were generated by the system, made it possible to obtain a fine-grained diagnostic of the developmental stage of the learners in their second language, while also providing information that is relevant to the implementation of a focused pedagogical intervention.

#### Integrating Dynamic Assessment in Computerized Adaptive Testing

Adaptive testing provides the opportunity to gain valuable insight into the challenges experienced by students with the items that compose a test depending on their degree of difficulty. It is also a valuable resource for gathering data about how each student approaches their response to each item as well as his or her skill level at the end of the test. However, analysis of these data cannot provide information on the type and degree of support that the subject requires to successfully answer a particular item. This information can only be obtained by integrating DA items, such as graduated prompts, feedback, or metacognitive guides, in the more general format of adaptive testing. This type of testing, which integrates DA and CAT techniques, makes it possible to obtain information not only about the response of a subject based on the difficulty of the test items, but also about the type and degree of support required to optimize performance and successfully solve the evaluated tasks. Thus, combining these assessment techniques could offer unique insights into the task resolution strategies adopted by students, which in turn could provide evidence about these students’ potential new knowledge and skills when receiving interventions that directly address their task-solving strategies. Given that CATs already offer the possibility of adapting the sequence of items in evaluation based on the skill level of the student, they provide an ideal setting for the implementation of DA.

In recent years, some CATs have included graduated prompts systems in their testing procedures as an assessment strategy in populations with learning difficulties, specifically those related to the development of reading skills (Stone and Davey, 2011; Petscher et al., 2016). Almost all of these assessment procedures have been created and marketed in the United States. Some notable examples are STAR-EL (Renaissance Learning; Shapiro, 2012) and the Children’s Progress Academic Assessment (CPAA, Northwest Evaluation Association; Bechard et al., 2010). STAR-EL comprises a set of computerized adaptive assessments in the areas of reading, math, and communication and includes a sophisticated system that provides specific support according to each student’s performance. The program also makes it possible to obtain reports of student performance and to compare a test with previous iterations of the test completed by the same student, tests of children under similar conditions, or standardized guidelines. McBride et al. (2010) showed that STAR-EL is technically suitable for schools and extremely convenient in terms of cost-benefit. CPAA is another computerized adaptive system that can be used three to six times during the year to monitor children’s progress. This system incorporates graduated prompts, which are taken into account in the calculation of the final student performance score. The program creates performance reports and offers specific guidance on the interpretation of the results for teaching.

### The Development of EDPL-BAI Battery

EDPL-BAI is a computerized assessment device that allows for the assessment of reading processes through a battery of tests that are delivered in a dynamic/adaptive format. It focuses on elementary school students, especially those with specific needs for support and learning difficulties in reading. It aims is to contribute to the psychoeducational evaluation of reading skills in the Spanish language. The EDPL-BAI includes various tests that tap into specific processes that are involved in reading and present different levels of difficulty, as well as a system of graduated prompts associated with each item of the test. Its essential feature is the dynamic adaptation of these elements to the level of competence progressively demonstrated by the student. The system adopts a quantitative and qualitative approach that encompasses both mediation and assessment processes. EDPL-BAI can record the sequence of actions followed by the student as well as the execution times for each one of these actions. In this regard, EDPL-BAI offers an individualized assessment while still establishing parameters based on IRT methods, which makes the results comparable with each other. The EDPL-BAI battery is composed of 38 adaptive tests grouped into six blocks of processes that are involved in reading: (a) underlying psychological processes and executive functions, (b) processes involved in grapheme–phoneme association, (c) lexico-morphological processes, (d) morpho-syntactic processes, (e) processes involved in the global comprehension of texts, and (f) personal-social adjustment processes.

EDPL-BAI is completed on a computer and is supported by the automatic evaluation web platform Siette ^1^, which allows the development and administration of the tests as well as collection and processing of data. This platform was used during the development of the IB, the calibration process, and the configuration of the graduated prompts for each item. The system allows users to combine, design, and manage tests from the viewpoint of classical test theory and IRT while also fostering the adaptive presentation of items, as recommended by CAT theory. The Siette system (Conejo et al., 2016) was developed by the Applications in Artificial Intelligence Research Group at the University of Malaga (Andalusia-Spain).

**FIGURE 3:**
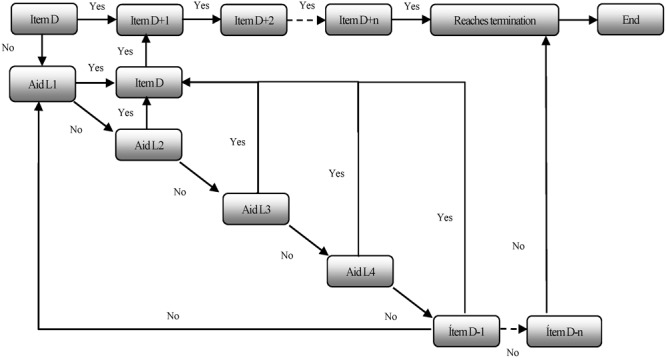
Sequence of actions and graduated prompts for each item in the EDPL-BAI. Item D, first item presented. Difficulty level D; Items D+1, D+2, items with higher degree of difficulty; Item D+n, item with the highest degree of difficulty; Item D-1, item with a lower degree of difficulty, which is introduced when the student fails the previous item after exhausting all the opportunities for support; Item D–n, item with the lowest degree of difficulty; Aids L1, L2, L3, and L4, support levels (the quantity and quality of aid increases).

The development of the EDPL-BAI battery followed five steps that are described below:

(1) *Creation of the IB and standard administration of the tests in Phase 1.* First, the items from the *Evaluación Dinámica de Procesos Lectores* [Dynamic Assessment of Reading Processes] (EDPL, Navarro et al., 2014) were adapted, and some additions were made to the original list of items. Once the IB was developed, we proceeded to apply it to a large sample of students (*n* = 1831) in a standard static format. In this way, based on the information obtained, it was possible to develop the validation and item calibration process based on IRT models. This process was necessary to later configure the battery in an adaptive mode.
(2) *Calibration process.* The calibration process allowed to adaptively configuring the items comprising each test based on the results obtained from the static administration of the tests. Different levels of difficulty associated with each item in each of the tests that make up the EDPL-BAI were empirically established. The calibration process also made it possible to associate the level of difficulty of each item with an estimate of the expected performance on each particular item by each student or group of students. The process was conducted using maximum likelihood (ML) methods, which estimate the parameters that maximize the likelihood of the observed responses and the level of knowledge of each student. The calibration process was carried out in several iterations in order to clarify the information and maximize the quality of the data that were obtained. The calibration process was performed on 1,336 items and more than 28,000 sessions, with less than 5% of invalid trials. MULTILOG was used for most of the calibration process, although JICS was also used in certain cases. A dichotomous model was established that included three parameters (3PL): (a) discrimination, (b) difficulty, and (c) guessing.
(3) *Mediation guidelines implementation.* For each item, we established specific mediation patterns in the form of graduated prompts. The establishment of these prompts was based on a qualitative analysis of the contents of each test. The aids were developed to be offered dynamically in reaction to the answers given by the students during the resolution of the tests. In general, up to four graduated prompt levels are offered. However, in some tests, a greater number of aids are offered, depending on the particular characteristics of the task. The general sequence of proposed prompts is as follows: (a) First support level (L1): *General prompts.* A general prompt is first proposed (e.g., “Read the instruction again; pay attention to the instruction; remember, chose the option that better answer the question”); (b) Second and third support level (L2 and L3): *Metacognitive prompts*. If the first level of assistance did not help, two levels of metacognitive prompts are then offered (e.g., “Read the sentence slowly and try to imagine it in your mind. Remember that some sentences are not plausible.” or “Read the sentence carefully and think about its meaning; then read carefully the question you must answer; when you have answered, review your answer carefully”); (c) Fourth support level (L4): *Specific prompts*. Finally, if none of the previous aids are helpful enough, specific support related to the construct under evaluation is proposed (e.g., “Pay attention to the question about who has done something. Look carefully at the choices and select the correct one…You must always look for who does something in the sentence”). Such differentiation is important because it makes it possible to clarify not only the amount or degree of aid required but also the type of aid that has been most effective for each student when responding to each item. At this point, it should be noted that the present paper only focused on the contribution of dynamic scores regarding the degree of support required. In any case, the inclusion of these graduated prompts during the assessment process allows for taking the subject’s response to mediation into account in the assessment process. **Figure 3** shows the specific sequence followed in each test once the graduated prompts were incorporated.
In line with the work of Guthke and Beckmann (2000), the proposed evaluation system envisages that students “move” through the various activities proposed without receiving aid at first. When a student fails to resolve an item, he or she is offered support related to the type of mistake that is expected for that type of item. Subsequently, the system provides items based on preestablished selection criteria. These criteria can be set based on the difficulty of the items. In such cases, when an error occurs, the system provides an item of equivalent complexity to that which caused the initial error, and if this second item is successfully resolved, the system presents other items with progressively higher levels of complexity. Alternatively, the selection criterion may be determined by the precision with which each item reports on the construct being evaluated.
(4) *Configuration of the computerized adaptive tests.* The criteria adopted in the configuration of the EDPL-BAI into an adaptive test were as follows: (1) *Initial trait level estimate*: Based on the scores obtained during the static testing phase for each one of the tests and each of the evaluated course levels (third, fourth, fifth, and sixth grade), an estimate of the level of difficulty for each item was computed. (2) *Completion criteria*: The completion criteria were established based on the most accurate information possible about the estimated current level of knowledge of the student. Indeed, the execution of each test had to be translated into a score of the estimated knowledge level of the student. For that, a scale from 1 to 7 –usually used in Chilean schools– was used. Based on this score, the next item that the student must complete was established. In principle, all tests terminate when one of the five values of the probability distribution of the estimated knowledge level reaches 80%, which is when the presented items explained 80% of the variance for estimation of knowledge level. (3) *The estimated level of knowledge*: A discrete distribution of student knowledge across five knowledge levels was established using the values obtained from item calibration. These estimated knowledge levels (𝜃), together with the values of the scale 1–7 to which they are associated are the following: very low (𝜃 = 1.60), low (𝜃 = 2.80), medium (𝜃 = 4.00), high (𝜃 = 5.20), and very high (𝜃 = 6.40). This categorization of knowledge level also makes it possible to determine the level of difficulty of each item in the context of each one of the knowledge levels. For example, an item may be challenging for students at the low and very low levels, but increasingly easy for students with a higher knowledge level.
In addition to the estimated score corresponding to the current level of student knowledge in each of the tests performed, which is estimated as a result of items calibrated based on the IRT, dynamic scores can be obtained through two heuristics. In one of them (the integrated dynamic score) the successes obtained without aid, the value is given to the aids that were effective, and the execution time is taken into account. The second establishes the dynamic score based on the inverse of the value of the required aids to successfully solve the items performed.
(5) *Configuration of the structure of the battery of tests.* The configuration of the internal structure of the set of tests that constitute the EDPL-BAI was determined by the consideration that various processes involved in reading require different skills and thus entail a different set of support needs and level of difficulty. In this regard, the general design of the internal structure of the EDPL-BAI was adjusted to the network of relationships established for the EDPL dynamic assessment device (Navarro et al., 2014). As mentioned previously, the original items were adjusted, expanded, and redefined during the first phase of the project to better address the specific issues that arise in the context of computerized adaptive assessment systems. Thus, the specific items that were shown to the student were then dynamically chosen within each block of reading processes based on the results of the calibration process.

### The Present Study: Objectives and Hypothesis

This article is part of more comprehensive research. The aim of the present study is to analyze the differential contribution on reading competence of the dynamic scores obtained from the implementation of a set of adaptive dynamic tests of morpho-syntactic processes integrated into the EDPL-BAI battery. Considering this objective, a structural equation model was implemented to check the relationship between the potentially predictive variables and criterion-reference tests. A model was built to test two hypotheses. The first one maintains that the dynamic scores would significantly relate to reading competence measured by the implementation of a standardized reading comprehension task and the teacher’s performance assessment (*Hypothesis* 1). Secondly, we expected to find a significant contribution of dynamic scores to the explained variance of both reading competence and teacher’s assessment. In this sense, we expect a dynamic score to signify an incremental explicative factor of reading competence in relation to the prediction based on the static tasks of non-verbal intelligence and comprehension (*Hypothesis* 2).

## Materials and Methods

### Participants

The research frame of this study involved 1831 students (46% girls) from 13 public schools in three regions of Chile (Metropolitan Region, Libertador Bernardo O’Higgins, and Araucanía). Non-probabilistic sampling was used. Initial contact was made with city councils of the three regions and through these, the public schools were accessed. Participation of the schools, teachers, and students was voluntary. The subsample selected for the present study initially consisted of 378 students belonging to six public urban and rural schools of the three regions. The students had completed the adaptive dynamic test of morpho-syntactic processes during Phase 2 of the project. From this sample, 54 students were removed based on their outlier performance on the tasks. The remaining 324 students (46% female) were in third (26), fourth (73), fifth (118), and sixth (107) grades and aged between 8 and 12 years old (*M* = 10.27, *SD* = 1.22).

### Instruments

#### Tests of the EDPL-BAI Battery (Morpho-Syntactic Processes)

##### Morpho-syntactic awareness test (MS) (Navarro and Rodríguez, 2014)

**FIGURE 4:**
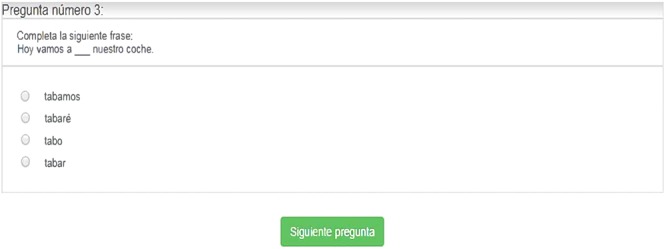
Item from the test of Morpho-syntactic Awareness (MS).

This test consists of 60 items. Each item is composed of a sentence that lacks a word or pseudoword (**Figure 4**). The student is presented with a sentence and asked to complete it using one of the words or pseudowords below [e.g., *Hoy vamos a (tabamos, tabaré, tabo, tabar) nuestro coche*]. Among the words that complete the sentences are the same number of nouns (derived morphology), verbs (inflectional morphology), and flexed pseudowords. All the words used are high frequency (frequency > 10), according to the Spanish Computerized Lexicon, LEXESP (Sebastián et al., 2000). Cronbach’s alpha for internal consistency for Morpho-syntactic Awareness Test (MS) (*N* = 261) = 0.99.

##### Syntactic awareness test

**FIGURE 5:**
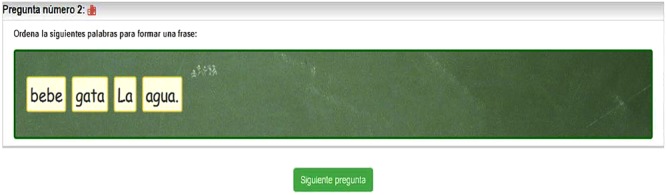
Item from the test of Syntactic Awareness (OF).

To sort disordered sentences (OF) (Navarro and Rodríguez, 2014). This test consists of 36 items. The items are composed of sentences that have been previously disordered. The student is presented with these sentences and asked to order them (moving the cards with the mouse on his or her computer) according to Spanish grammar rules (**Figure 5**). Half of the sentences are semantically plausible once ordered; that is, they express ideas or familiar situations (*bebe gata La agua. [drinks cat The water.]*), and the other half are implausible (*La novelas. lee mesa [The novels. reads table]*). The sentences also differ in length (short/long) and syntactic complexity (active/passive, simple/compound, coordinated/subordinate). All the words used are high frequency (frequency > 10), according to the Spanish Computerized Lexicon, LEXESP (Sebastián et al., 2000). The test consists of 24 simple sentences and 12 compound sentences. Among the simple ones, four are short (< five words), all of which are active, and 20 are long (> six words), 16 of which are active and four passive/reflexive. Among the compound sentences (all of them long), four are coordinated and eight are subordinate (relative and conditional). The sentences are shown disordered, but each word is accompanied by the punctuation mark with which it appears in the original sentence, as in the example in **Figure 5**. Likewise, the first word of the original sentence maintains the initial capital letter when the phrase is disordered, as we can see in the previous example. Cronbach’s alpha for internal consistency for Syntactic Awareness Test (OF) (*N* = 323) = 0.88.

##### *Syntactic awareness test* (CS) (Navarro and Rodríguez, 2014)

**FIGURE 6:**
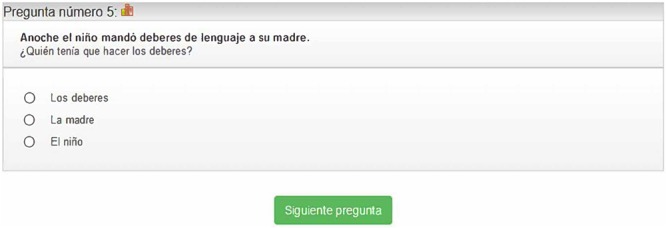
Item from the test of Syntactic Awareness (CS).

This test consists of 44 items. It is based on a test designed by Miller (2010) with the same purpose. The CS test evaluates syntactic awareness based on the student’s understanding of sentences and answers to questions that contain different syntactic keys. In each item the student is presented with a sentence and then a question referring to one of the characters or elements that have appeared in the sentence (**Figure 6**). The student must choose the correct answer among the three options. All the sentences are formed by words with a frequency greater than 10 according to the LEXESP (Sebastián et al., 2000). Of the total number of sentences, 28 are simple and 16 are compound. Of the simple sentences, 20 are active/transitive and eight are passive. Half of the questions are directed to the person who performs the action (subject), and the other half to the person who receives the action (object). On the other hand, in the compound sentences, there are eight coordinates and eight subordinates. Half of the sentences are plausible (they correspond to possible events), and the other half are implausible (they correspond to impossible facts or facts that contradict our previous knowledge). The correct option is counterbalanced. In the semantically implausible sentences, it is not possible to access the meaning (and thereby solve the task adequately) without performing syntactic processing. Cronbach’s alpha for internal consistency for Syntactic Awareness Test (CS) (*N* = 257) = 0.76.

The sequence of test items in the EDPL-BAI battery was presented adaptively and dynamically. The selection of the initial item was based on the student’s grade average performance. Later, during the administration, the selection of the items was based on the answers and the student’s performance. If a student made a mistake, aid was offered. As mentioned before, four levels of graduated prompts were included for each item. The graduation ranged from more general to more specific aids. To obtain a weighted value of the aids, a value of 4 was given to the most general aids and 1 to the most specific ones. The items that were solved without aid received 5 points.

#### Criteria Measures

##### Reading comprehension tests CLPT (Medina et al., 2010)

The tests evaluate different dimensions involved in reading comprehension and the writing of texts. The test has specific forms for grades third to sixth with 16 items each. In this study, only reading comprehension was assessed. The CLPT was administered twice (CLPT_2_Pre and CLPT_Post, respectively) to explore the predictive and incremental validity of dynamic scores. The administration was conducted in the classrooms. The CLPT tests have been validated in Chile and are widely used in the school settings. The CLPT tests were designed based on an updated review of scientific literature of reading comprehension. The authors report acceptable values of corrected item-total correlation indexes. Also, test–retest reliability is considered acceptable. Cronbach’s alpha for internal consistency was analyzed with data of Phase 1 in the present study. The results for each grade were the following: alpha third (*N* = 371) = 0.71; alpha fourth (*N* = 390) = 0.78; alpha fifth (*N* = 351) = 0.57; alpha sixth (*N* = 363) = 0.51.

##### Pretest and posttest EDPL-BAI

Two tests were created to be administered as a pretest and posttest of the EDPL-BAI battery. Each of these tests consisted of 80 items, which were selected based on the items that are part of the battery tests. The items selected and extracted for the pre- and posttest were equivalent in difficulty and discrimination indexes. The pretest and posttest each included 16 items from the pseudoword reading test, 16 items from the word reading test, 12 items from the morphological awareness test, 12 items from the Morpho-syntactic Awareness test, 10 items from the Syntactic Awareness test (CS), 6 items from the Syntactic Awareness test (OF), and 8 items from the text comprehension test. The percentage of correct responses was used as a measure of performance in these tests (Por_Pre and Por_Post2, respectively). Cronbach’s alpha for internal consistency for *Pretest EDPL-BAI* (*N* = 820) = 0.88, and for *Posttest* (*N* = 736) = 0.99.

##### Test of Raven’s progressive matrices (Raven, 1995)

This test is used to evaluate analogical reasoning skills with the aim of obtaining information regarding students’ cognitive performance. Using this test in the present study provides a measure of non-verbal intelligence to control its specific contribution on reading competence. The general scale, applied from the fourth to sixth grade, consists of 60 items, and the colored scale, which applies to younger students and students who present special educational needs, consists of 36 items. The *z* scores were computed independently for each grade and used for the analysis.

##### Teachers’ assessment of reading performance (Valor_Prof)

Once the application was completed, the teacher had to use a qualitative scale to assess seven specific evaluation criteria formulated in relation to the reading processes contemplated in the tasks, as well as in the criteria proposed in the Curricular Bases for Language. The qualitative grading applied to each of the criteria of the template was as follows: (1) low-very low level, (2) medium-low level, (3) average level, (4) medium-high level, and (5) high-very high level. The evaluation criteria were (a) he/she reads and understands different types of school texts appropriate to his/her grade, highlighting the topic and main ideas; (b) he/she makes diagrams or summaries in a clear and orderly manner, capturing the overall meaning of the text; (c) he/she extracts data and information from graphs and tables, using it in the resolution of problems/activities appropriate to his/her course; (d) he/she integrates explicit and implicit information and makes inferences based on elements of the text and also on previous knowledge; (e) he/she raises doubts or asks questions when reading, realizes when he/she does not understand something, and rereads when he/she has not understood the text; (f) he/she expresses his/her opinion, comments on the text already read, makes judgments, or proposes solutions to problems raised in the read texts; and (g) he/she perceives him/herself to be effective and competent when faced with reading activities and shows a positive attitude toward reading. This measure was used as an external criterion of the teacher’s assessment of the performance observed during the administration period (Resing, 2000; Caffrey et al., 2008). The average score for the seven criteria was used for the analysis.

### Procedure

After the items calibration was addressed during Phase 1, the administration of criteria tests and of the EDPL-BAI was conducted during Phase 2. The implementation of the criteria tests as well as the EDPL-BAI battery tests during this phase of the study was carried out in educational centers by research assistants, who received training related to the theoretical/methodological bases of the proposal. These were postgraduate or final-year students of pedagogy or psychology. The administration of tests was collectively carried out (in class groups), in the usual educational context of the students. Each student received a total of 8 sessions: one session of 75 min for the CLPT pre-test, one session of 45 min for the administration of the Raven test, one session of 45–60 min for the EDPL-BAI pretest, four sessions of 75 min each for the administration of the EDPL-BAI battery, and one session of 45–60 min for the EDPL-BAI posttest. After 4–5 months, each student received two tests: the CLPT posttest, and the EDPL-BAI posttest. Likewise, a total of 12 teachers collaborated in the completion of the rating scales on reading performance.

Regarding ethical considerations, this study was carried out following the recommendations of the Declaration of Helsinki. The protocol was approved by the Scientific Ethics Committee of the Universidad Autónoma de Chile, Santiago, Chile. All subjects gave written informed consent in accordance with the Declaration of Helsinki. Before data collection, consent was obtained from the students’ families and the students, informing them of the conditions of confidentiality and administration of the tests.

### Design and Data Analysis

A correlational research design based on causal models was proposed. It aimed to determine the incremental validity of dynamic scores on students’ reading competence in relation to predictions based on static measures of comprehension and non-verbal intelligence. Dynamic scores were obtained from the implementation of the tests of the EDPL-BAI battery. On the one hand, the *student’s knowledge level* was estimated from the previous items calibration process. In this study, the calibrated score was calculated as the mean of the distribution obtained by the iterative process which represented in **Figure 1**. On the other hand, as mentioned before, two heuristics were used: the integrated dynamic score (IDS), and the dynamic score based on the inverse of the value of the required aids to successfully solve the items performed (DS_Inv). In this study, only this second heuristic is reported. The formula for computing this dynamic score is as follows:

$$\begin{array}{c} DS\_Inv\;=\;(TCR^{*}10)\;-\;VRA \end{array}$$

where TCR is the total correct responses in the test, with and without aids; 10 is the sum of the values assigned to the aids in each item (Aid L1 = 4 + Aid L2 = 3 + Aid L3 = 2 + Aid L4 = 1); and VRA is the value assigned to the required aids during the test execution.

The descriptive statistics were obtained to characterize the sample performance in all the measures. The outliers were explored using box plots with a labeling rule of 2, 2 (Hoaglin et al., 1986) and visual exploration of technical errors in the data recording. The correlation matrix was examined to explore the relationship between the measures as well as the concurrent and predictive validity. An initial structural equation model was built to test the hypothesized relationship between the measures as well as to test the incremental validity of the EDPL-BAI battery over the criteria test. The model included four potential predictive variables: (a) DA factor made of the dynamic scores from MS, OF, and CS tests; (b) the *z* scores from Raven test; (c) the EDPL-BAI pretest, and (d) the CLPT pretest. All these variables are related to each other. Then the predicted variables were: (a) CLPT posttest; (b) EDPL-BAI posttest and; (c) Teachers’ assessment of reading performance. All the predicted variables were assumed to be correlated. Using this model as a template, two different models were explored. In the Model 1 the DA factor was made of the calibrated scores, and the Model 2 explored the dynamic scores based on the inverse of the value of the required aids. For the two models the non-significant paths were deleted sequentially.

Regarding the assumption of normality, the analysis showed an absence of normality, with values of asymmetry and kurtosis that exceeded the established limit of ±1.96 (*p* < 0.05). Thus, considering the lack of multivariate normality of some variables in the model the asymptotically distribution-free method was used to estimate the parameters (Browne, 1984). The indices used to assess goodness-of-fit of each model were: the χ^2^ (*p* = 0.05 or greater indicating an appropriate fit); a ratio of χ^2^/*df* (χ^2^/*df* < 3, appropriate fit); Comparative Fit Index (CFI) (CFI ≥ 0.95 appropriate fit); Root Mean Square Error of Approximation (RMSEA) (RMSEA ≤ 0.06 indicating a good fit of the model) (Byrne, 2016). The statistical analysis was conducted using the programs SPSS-22 and AMOS-25 (Inc, Chicago, IL, United States). Statistical significance was set at *p* < 0.05.

## Results

### Descriptive Results

**Table 1** shows the descriptive statistics of the values and scores obtained in the study. The differences in the sample size was due, on the one hand, to the cases in which the students could not complete all the tests administered during the study, and on the other, to the fact that the characteristic of adaptability in relation to the tests that each student took based on their answers, caused that not all students go through the exact same tests.

**Table 1:** Descriptive statistics of the values and scores obtained.

Variable	*N*	Media	*SE*	*SD*	95% CI	
					Lower limit	Upper limit
Por_Pre (80)	248	69.12	0.77	12.18	67.60	70.65
Por_Post2 (80)	214	70.58	0.89	12.98	68.83	72.33
CLPT_pre	261	28.93	0.58	9.43	27.78	30.08
CLPT_post	186	31.95	0.72	9.77	30.54	33.37
Nivel_CS (1–7)	199	4.66	0.06	0.82	4.54	4.77
Nivel_MS (1–7)	196	4.46	0.06	0.90	4.33	4.59
Nivel_OF (1–7)	283	3.98	0.07	1.16	3.85	4.12
DS_Inv_CS	199	68.56	1.43	20.16	65.74	71.38
DS_Inv_MS	196	75.86	1.78	24.90	72.35	79.37
DS_Inv_OF	283	29.32	0.93	15.71	27.48	31.16
Z_Raven	218	0.16	0.07	0.97	0.04	0.29
Valor_Prof (1–5)	212	3.56	0.06	0.94	3.43	3.68

*SD, standard deviation; SE, standard error of the mean; CI, confidence interval of the mean; Por_pre and Por_post2, percentage of correct answers in the pretest and posttest of EDPL-BAI; CLPT_pre and CLPT_post, pretest and posttest scores in the CLPT test; CS, dynamic Syntactic Awareness test; MS, dynamic Morpho-syntactic Awareness test; OF, dynamic Syntactic Awareness test (order sentences); DS_Inv, dynamic score obtained from the inverse of the value of the required aids; Z_Raven, punctuation typified in the Raven test; Valor_Prof, average of the teacher’s assessment (1–5) on reading performance.*

The mean values of the two dynamic scores computed under different methods were consistent to show that the OF task was more difficult for the students, while the performance on MS and CS were similar.

### Correlation Results Between the Values and Scores Obtained

**Table 2** shows the correlation coefficients obtained and their level of significance. It is possible to observe significant levels of correlation between the pretest and posttest scores of the CLPT criterion test. These levels were expected and confirmed the validity of this test for the purposes we have used it for. Regarding the concurrent validity, moderate correlation coefficient were observed between the dynamic scores and the CLPT pretest. Likewise, dynamic scores significantly correlated with the static criterion measures, the CLPT posttest, the teacher’s assessment of reading performance, and the EDPL-BAI posttest. This occurs for the calibrated score (Nivel), which reflects the estimated student’s knowledge level, and also for the dynamic scores based on the inverse of the value of the required aids (DS_Inv).

**Table 2:** Correlation coefficients (Pearson) between the different values and scores obtained on the tests administered.

	1	2	3	4	5	6	7	8	9	10	11	12
1. Por_Pre	1											
2. Por_Post2	0.61^∗∗∗^	1										
3. Nivel_CS	0.55^∗∗∗^	0.52^∗∗∗^	1									
4. Nivel_MS	0.59^∗∗∗^	0.57^∗∗∗^	0.60^∗∗∗^	1								
5. Nivel_OF	0.45^∗∗∗^	0.53^∗∗∗^	0.37^∗∗∗^	0.41^∗∗∗^	1							
6. DS_Inv_CS	0.58^∗∗∗^	0.50^∗∗∗^	0.87^∗∗∗^	0.60^∗∗∗^	0.41^∗∗∗^	1						
7. DS_Inv_MS	0.58^∗∗∗^	0.56^∗∗∗^	0.63^∗∗∗^	0.88^∗∗∗^	0.44^∗∗∗^	0.63^∗∗∗^	1					
8. DS_Inv_OF	0.42^∗∗∗^	0.41^∗∗∗^	0.39^∗∗∗^	0.40^∗∗∗^	0.79^∗∗∗^	0.39^∗∗∗^	0.43^∗∗∗^	1				
9. CLPT_2_pre	0.25^∗∗∗^	0.39^∗∗∗^	0.34^∗∗∗^	0.35^∗∗∗^	0.26^∗∗∗^	0.36^∗∗∗^	0.32^∗∗∗^	0.18^∗∗^	1			
10. CLPT_post	0.26^∗∗∗^	0.35^∗∗∗^	0.32^∗∗∗^	0.41^∗∗∗^	0.24^∗∗∗^	0.36^∗∗∗^	0.38^∗∗∗^	0.13	0.53^∗∗∗^	1		
11. Valor_Prof	0.37^∗∗∗^	0.23^∗∗^	0.44^∗∗∗^	0.37^∗∗∗^	0.26^∗∗∗^	0.44^∗∗∗^	0.45^∗∗∗^	0.29^∗∗∗^	0.42^∗∗∗^	0.35^∗∗∗^	1	.
12. Z_Raven	0.42^∗∗∗^	0.40^∗∗∗^	0.31^∗∗∗^	0.26^∗∗^	0.38^∗∗∗^	0.32^∗∗∗^	0.23^∗∗^	0.27^∗∗∗^	0.19^∗∗^	0.27^∗∗^	0.21^∗∗^	1

*Por_pre and Por_post2, percentage of correct answers in the pretest and posttest of EDPL-BAI; CLPT_pre and CLPT_post, pretest and posttest scores in the CLPT test; CS, dynamic Syntactic Awareness test; MS, dynamic Morpho-syntactic Awareness test; OF, dynamic Syntactic Awareness test (order sentences); DS_Inv, dynamic score obtained from the inverse of the value of the required aids; Z_Raven, punctuation typified in the Raven test; Valor_Prof, average of the teacher’s assessment (1–5) on reading performance. ^∗^*p* < 0.05; ^∗∗^*p* < 0.01; ^∗∗∗^*p* < 0.001.*

### Theoretical Structure of the Causal Model

**FIGURE 7:**
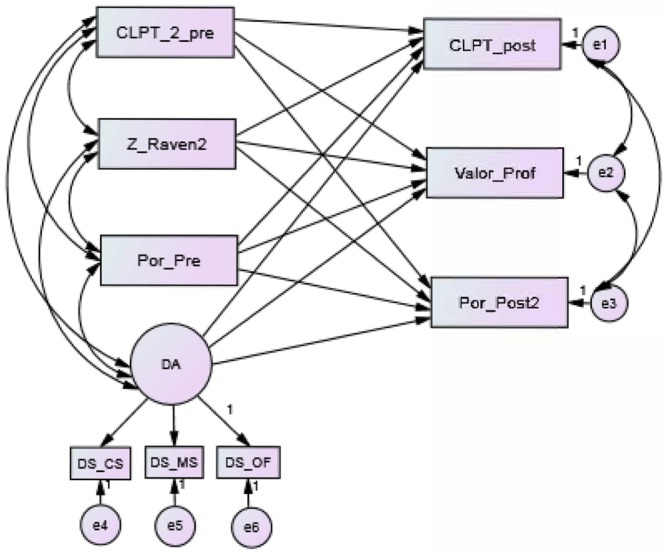
Design of theoretical structural equation modeling. CLPT_pre, pretest scores in the CL-PT test; Z_Raven, punctuation typified in the Raven test; Por_pre, percentage of correct answers in the pretest of EDPL-BAI; DA, latent variable refers to dynamic scores obtained from EDPL-BAI; CS, dynamic Syntactic Awareness test; MS, dynamic Morpho-syntactic Awareness test; OF, dynamic Syntactic Awareness test (order sentences); CLPT_post, posttest scores in the CL-PT test; Valor_Prof, average of the teacher’s assessment (1–5) on reading performance; Por_post2, percentage of correct answers in the posttest of EDPL-BAI.

According to the objectives and hypotheses formulated, it is necessary to determine the differential contribution of the scores obtained on the dynamic tests of EDPL-BAI battery on reading competence, as measured by both the static posttests and the quantified teacher’s assessment on reading performance. For this, a causal theoretical model is proposed for contrasting the data. This model (**Figure 7**) was developed to check the extent to which DA, the latent variable measured by the three dynamic tests, can explain the reading competence, taking into joint consideration the magnitude of the contributions produced by the static measures of reading comprehension and non-verbal intelligence. In this sense, the theoretical model presents, on the one hand, the rest of the predictor variables, all of which are observed variables (CLPT_2_pre, Z_Raven2, and Por_Pre), and on the other hand, the variables used as a criterion, which are all observed variables as well (CLPT_post, Value_Prof, and Por_Post2). The time elapsed between the two moments was 5 months. Among the predictor variables, we included the different dynamic scores mentioned previously in successive analyses.

### Structure and Standardized Solution to the Model 1

#### Contrasting the Analysis Model With Previous Assumptions

As a previous step to the analysis made with the structural equation model, several tests were carried out to verify the multivariate normality assumptions and model identification. First, with respect to the assumption of normality, the asymmetry and kurtosis values of the observed variables were analyzed. The analysis showed that five of the nine values of asymmetry (all negative) indicated an absence of normality in the distribution. These values (critical ratio) were below -1.96 (*p* < 0.05). With respect to kurtosis, four values (all positive) also indicated an absence of normality. In these cases, the values exceeded 1.96. Taking these data into account, together with the results of the Kolmogorov–Smirnov univariate normality test, in which eight of the nine variables did not fulfill the normality assumption (*p* < 0.001), it was decided to apply an asymptotically distribution-free estimation method (Browne, 1984). In relation to the model identification, the results of the analyses show that it is an identified model. In this sense, the order condition was verified (18 degrees of freedom) as well as the condition range (assuming that the covariance matrix is positively defined, the determinant of the covariance matrix departs substantially from the value 0). We also found a lack of variance/covariance negative error, excessively high standard errors, and correlations between estimated coefficients above 0.80.

#### Checking the Model Fit

First, we present the evaluation of the model adjustment, taking into consideration the score that reflects each student’s estimated knowledge level—that is, the score offered by the system, which is calculated based on the items previously calibrated. Global and comparative adjustment indexes have been taken into account. Likewise, we also proceeded to introduce parsimonious adjustment measures, which provide information about the simplicity of the model. It should be noted that the application of non-parametric techniques for the estimation of parameters could lead to lower efficiency in the specification of the model, which could affect the scope of the proposed model. In relation to the adjustment of the global model, **Table 3** shows several indicators. First, the chi-square contrast shows an adequate global adjustment value (*p*-value = 0.161). For its part, the root mean square error of approximation indicator shows an acceptable value (RMSEA = 0.032). With respect to the CFI and TLI indexes, they show optimal values close to 1. Similarly, the adjusted parsimony measures show values that are within the acceptable range: PRATIO = 0.500 and PCFI = 0.492. Finally, we can see that the model explains 56% of the variance in reading comprehension, measured with the posttest of the static test CLPT_post, 46% of the variance in reading performance measured by the teacher’s assessment, and 68% of the variance in reading performance, measured by the EDPL-BAI posttest.

**Table 3:** Indicators of fit for Model 1.

χ^2^	*df*	*p*	CFI	TLI	RMSEA	PRATIO	PCFI	*R*^2^	
23.830	18	0.161	0.983	0.966	0.032	0.500	0.492	CLPT_post	0.56
								Valor_Prof	0.46
								Por_Post2	0.68

*CFI, Comparative Fit Index; TLI, Tucker-Lewis coefficient; RMSEA, Root Mean Square Error of Approximation; PRATIO, Parsimony Ratio; PCFI, parsimony fit to the CFI.*

#### Analysis of Individual Relationships

**FIGURE 8:**
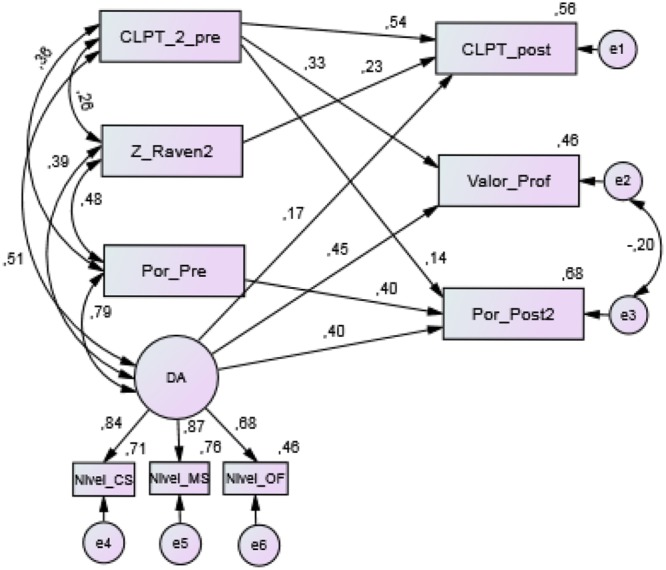
Standardized solution to the Model 1. CLPT_pre, pretest scores in the CL-PT test; Z_Raven, punctuation typified in the Raven test; Por_pre, percentage of correct answers in the pretest of EDPL-BAI; DA, latent variable refers to dynamic scores obtained from EDPL-BAI; Nivel, estimated student’s knowledge level; CS, dynamic Syntactic Awareness test; MS, dynamic Morpho-syntactic Awareness test; OF, dynamic Syntactic Awareness test (order sentences); CLPT_post, posttest scores in the CL-PT test; Valor_Prof, average of the teacher’s assessment (1–5) on reading performance; Por_post2, percentage of correct answers in the posttest of EDPL-BAI.

An individual analysis of the regression coefficients for each of the routes proposed in the model was carried out (**Figure 8**). In this sense, the standardized solution shows significant relationships between the variables at a significance level of α = 0.001. Also, both the covariance and the correlations between the exogenous (independent) variables are significant (*p* < 0.001). Regarding the observed exogenous variables, the highest contribution (0.54) is produced by the CLPT pretest (CLPT_2_pre) on the score obtained on the posttest of the same test, while the contribution of the non-verbal intelligence test is 0.23. On the other hand, the adaptive/dynamic administration of the morpho-syntactic tests (DA) shows a significant and incremental contribution on the three criteria variables (0.17 on the CLPT posttest, 0.45 on reading performance as evaluated by the teachers, and 0.40 on the EDPL-BAI battery post-test).

### Structure and Standardized Solution to the Model 2

As mentioned before, the analyses to evaluate the model adjustment were replicated by introducing the dynamic scores that had been calculated as a result of the implementation of the tests. In all the analyses performed, the previous assumptions of multivariate normality and model identification were contrasted. Depending on the interest of the contributions analysis of the scores derived from the calculation of heuristics, we collect the standardized solution for the dynamic scores based on the inverse of the value of graduated aids required by the student during the task resolution. In this sense, concerning the assumption of normality, the analysis showed an absence of normality, with values of asymmetry and kurtosis that exceeded the established limit of ±1.96 (*p* < 0.05). Therefore, as in the previous case, we decided to apply an asymptotically distribution-free estimation method. With respect to the identification of the model, the results of the analyses show that it also was an identified model.

**Table 4** shows the indicators obtained in relation to the evaluation of the model’s global adjustment. The chi-square contrast shows an acceptable value (*p*-value = 0.073), as does the RMSEA indicator (0.039). With respect to the other indicators evaluated, the analysis related to the CFI index shows a value close to 1, which indicates a good level of adjustment, and the TLI index also shows an acceptable value. Likewise, the adjusted parsimony measures show values that are within the acceptable range: PRATIO = 0.528 and PCFI = 0.513. Finally, we can see that the model explains 53% of the variance in the posttest of CLPT, 49% of the variance in reading performance measured with the teacher’s assessment, and 66% of the variance in the EDPL-BAI posttest.

**Table 4:** Indicators of fit for Model 2.

χ^2^	*df*	*p*	CFI	TLI	RMSEA	PRATIO	PCFI	*R*^2^	
28.562	19	0.073	0.972	0.948	0.039	0.528	0.513	CLPT_post	0.53
								Valor_Prof	0.49
								Por_Post2	0.66

*CFI, Comparative Fit Index; TLI, Tucker-Lewis coefficient; RMSEA, Root Mean Square Error of Approximation; PRATIO, Parsimony Ratio; PCFI, parsimony fit to the CFI.*

#### Analysis of Individual Relationships

**FIGURE 9:**
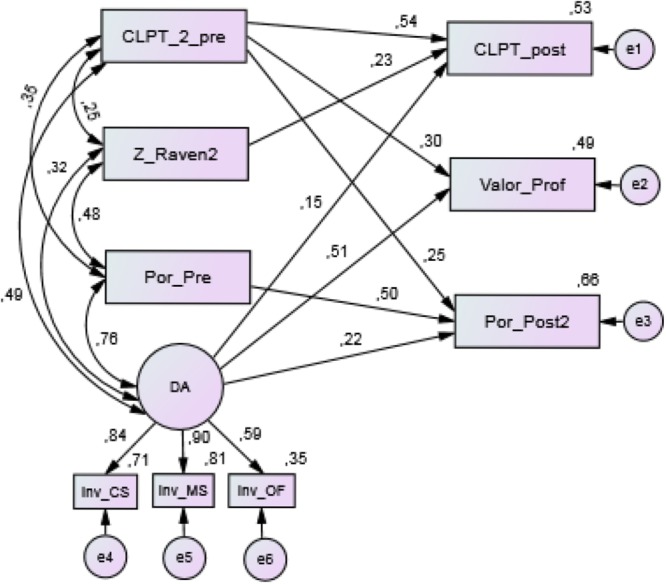
Standardized solution to the Model 2. CLPT_pre, pretest scores in the CL-PT test; Z_Raven, punctuation typified in the Raven test; Por_pre, percentage of correct answers in the pretest of EDPL-BAI; DA, latent variable refers to dynamic scores obtained from EDPL-BAI; DS_Inv, dynamic score obtained from the inverse of the value of the required aids; CS, dynamic Syntactic Awareness test; MS, dynamic Morpho-syntactic Awareness test; OF, dynamic Syntactic Awareness test (order sentences); CLPT_post, posttest scores in the CL-PT test; Valor_Prof, average of the teacher’s assessment (1–5) on reading performance; Por_post2, percentage of correct answers in the posttest of EDPL-BAI.

In this case, we also carried out an individual analysis of the regression coefficients for each of the routes proposed in the model (**Figure 9**). The model’s standardized solution shows significant relationships between the variables at a significance level of α = 0.001, except for two variables, which show a significance level of α = 0.01. Also, both the covariance and the correlations between the exogenous (independent) variables are significant (*p* < 0.001). Regarding the observed exogenous variables, the highest contribution is still the one produced by the CLPT_2_pre (0.54) score on the CLPT posttest. The adaptive/dynamic application of the morpho-syntactic tests (DA) shows a significant and incremental contribution on the reading performance as evaluated by the teachers (0.51), on the CLPT posttest (0.15), and on the EDPL-BAI posttest (0.22).

## Discussion

One of the main objectives with regard to the development and implementation of psychoeducational evaluation models is that they offer valuable information oriented to the intervention. The computerized adaptive DA seeks to offer incremental information to that provided by conventional tests (Sternberg and Grigorenko, 2002). This incremental information is specified in providing data on the task resolution process, including the difficulties encountered by the student during the task, as well as data related to the analysis of the established mediation process. In this sense, the study of the mechanisms of action that can explain the improvements that can be observed during the implementation of DA procedures, and through which a student would optimize his or her learning, can be significant for inferring subsequent intervention guidelines (Grigorenko, 2009). The present study aimed to analyze the contribution on reading competence of the three adaptive dynamic tests that make up the block of morpho-syntactic processes of the EDPL-BAI battery. In this sense, two specific hypotheses were formulated. The first hypothesis argued that the dynamic scores obtained from the EDPL-BAI would be significantly related to the measures of subsequent performance in reading. The second one held that dynamic scores would contribute significantly to explain the variability in reading competence and would constitute an additional explanatory factor of reading performance over the static tests of non-verbal intelligence and reading comprehension.

With respect to the first hypothesis, we must emphasize that the dynamic scores establish a significant relationship with the standardized reading comprehension test and the qualitative teacher’s assessment. In this sense, the teacher’s assessment allows the introduction of evaluative elements of a procedural nature that can hardly be evaluated with static performance measures. Likewise, the teacher’s assessment has proven to be a good predictor of academic performance (Navarro and Mora, 2013). Also, the evaluation of the reading performance by teachers could increase the ecological validity of the results, since the assessment corresponds to the level of demand that is considered essential by the professionals in charge of the teaching-learning process.

Regarding the second hypothesis, the results obtained show that the dynamic scores obtained from the application of the EDPL-BAI battery further explain the variability in reading competence as measured with the CLPT test, the EDPL-BAI posttest, and the teacher’s assessment of reading performance. In this sense, the analysis of the regression coefficients of the model’s standardized solution indicates that the dynamic application of the tests maintains a significant and incremental contribution on the three measures of reading competence once the rest of the predictor variables are controlled for. This was observed for both the estimated student’s knowledge level and the dynamic score obtained from the inverse of the value of the required aids.

One of the central questions that must be answered by an intervention-oriented DA approach is to know what the incremental proportion of variance explained by the dynamic scores represents. The incremental validity means that a part of the variance of the criterion measures can be explained as a result of the information that is derived from the application of the dynamic tests. In this sense, an analysis of the elements that can explain the changes could offer valuable information about the functioning of the subject. In particular, in the context of DA, this analysis of change is aimed at establishing what the subject is capable of performing when offered guidelines and graduated prompts—that is, informing us of his or her learning potential (King et al., 2015; Poehner et al., 2015). Therefore, the incremental proportion of variance explained by the dynamic scores would represent a quantification of learning potential. This change potential is reflected in the development of competencies that facilitate learning and benefit from the mediation offered—in this case, in the form of the graduated prompts. It is important to note that these aspects are not evaluated in the conventional tests. Likewise, in accordance with the idea to establish those mechanisms of action that could optimize the learning process, the information obtained through the dynamic application of the tests is qualitatively different from what we could obtain with the application of a standard comprehension test. This additional information would be mainly about the difficulties that the student manifests during the task resolution process, as well as about the mediation guidelines that are effective during the test administration process.

In line with the results obtained by King et al. (2015), and Hasson et al. (2012), the implementation of DA tests would have provided valuable information regarding the process followed by the students during the task resolution. This information would contain, in our case, data on the aids that were most effective in successfully resolving the different items, which might be useful regarding understanding the difficulties and the ways of intervening to resolve them. In this sense, the results of the model that introduces the dynamic scores based on the inverse of the value of the required aids show that those students who needed more aids, and especially aids specifically related to the difficulties of the task, obtained worse results in the criteria measures. The next step in an intervention-oriented evaluation procedure is obviously to qualitatively analyze the specific aids required that were effective in successfully resolving the items, with the aim of guiding the educational intervention and contributing to the improvement of the subject’s functioning.

Like Poehner et al. (2015) and Kozulin and Garb (2002), we used heuristics to obtain differential information from the score that included data on the required aids. In our case, the dynamic scores that comprised the graduated prompts are not yet automatically generated by the system, but we expected that obtaining them would offer us information on the amount and type of aid that would be effective. Although we have presented only the standardized solution of the model that included the dynamic scores based on the inverse of the value assigned to the aids required to successfully solve the items carried out, the rest of the calculated scores (between them the heuristics used by the authors cited above) have shown, with some differences, significant contributions on the measures of reader performance. These scores also made it possible to obtain incemental information regarding the assessment of the difficulties in the reading processes analyzed, while providing relevant information oriented to the intervention.

### Limitations of the Study and Future Analysis and Development

The present study offers information about the implementation of three adaptive dynamic tests of the EDPL-BAI battery. However, the battery has 38 tests that integrate six blocks of processes involved in reading. The research that supports the present study is still under development, so the data shown represent only a part of the sample. Likewise, we must mention that the longitudinal nature of the study, developed over the course of 2 years, required the continuous collaboration of the educational centers. In this sense, despite this collaboration, it was not possible to obtain all measures for all participants. Another consideration that we should point out is that, although the research assistants who participated in the group implementation of the EDPL-BAI battery received specific training, the logistical and technical difficulties encountered during the implementation in the schools sometimes made data collection difficult. Also, we must point out some problems related to connectivity or access to the online evaluation platform, the availability of computers in computer labs in schools, and the availability of network support in rural schools. Finally, the system of graduated prompts used in Phase 2 could not be fully implemented, since they only appeared in audio format and not in both, audio and writing format.

Regarding future analysis, the differential validity of dynamic scores on reading performance for different subgroups of students must be analyzed. These analyses could establish differences about the conditions of greater efficiency and effectiveness in the use of the EDPL-BAI battery, as well as in relation to the information that can be obtained (Navarro and Mora, 2013). Likewise, future analyses must also differentiate the results by grade or by different age groups.

Among the main advantages that derive from the use of computerized adaptive assessment tools, there is the possibility of continuously improving the different elements that make up the device, e.g., adjustments to the test interface, the item bank, the calibration process, or the system of graduated prompts. In this sense, the complete development of the system of graduated prompts, together with the joint calibration of the items and the aids, will result in greater possibilities for obtaining optimal results about predictive and incremental validity, and especially about the analysis of the effectiveness of the aids. Likewise, in relation to the users, the use of CAT based on IRT models that also incorporate a system of graduated prompts offers a series of additional advantages: it allows for recording and analyzing the sequence of actions performed by the student, the execution time, the successes and errors based on the attempts made, the difficulty levels of each item, and the aids required to successfully solve the items. The possibility of having all these data and of analyzing them in an integrated way could offer valuable information oriented to the intervention and improvement of the evaluated processes. Specifically, the incorporation of graduated prompts into computerized adaptive assessment models would offers control and activity regulation tools and allow observation and assessment of the degree of incorporation of these tools by the student during the task resolution.

## Author Contributions

J-JN and CM-C conceived and designed the study. J-JN, CM-C, and IR-O performed tables and figures. CM-C and J-JN analyzed the data. EG, IR-O, CM-C, RC, CS-G, JdlF, J-JN, DM, and MS contributed materials and analysis tools. J-JN, CM-C, EG, CS-G, and IR-O wrote the paper. IR-O, CM-C, CS-G, JdlF, EG, RC, DM, and MS paper revision.

## Conflict of Interest Statement

The authors declare that the research was conducted in the absence of any commercial or financial relationships that could be construed as a potential conflict of interest.

## Footnotes

1 www.siette.org
